# l-DOPA and consolidation of fear extinction learning among women with posttraumatic stress disorder

**DOI:** 10.1038/s41398-020-00975-3

**Published:** 2020-08-15

**Authors:** Josh M. Cisler, Anthony A. Privratsky, Anneliis Sartin-Tarm, Kyrie Sellnow, Marisa Ross, Shelby Weaver, Emily Hahn, Ryan J. Herringa, George Andrew James, Clinton D. Kilts

**Affiliations:** 1 Department of Psychiatry, University of Wisconsin Madison, Madison, WI USA; 2 University of Arkansas for Medical Sciences, Brain Imaging Research Center, Little Rock, AR USA; 3 Massachusetts General Hospital/Harvard Medical School, Boston, MA USA

**Keywords:** Neuroscience, Learning and memory

## Abstract

This study tested whether l-DOPA delivered during the consolidation window following fear extinction learning reduces subsequent fear responding among women with PTSD. Adult women diagnosed with PTSD completed a contextual fear acquisition and extinction task during fMRI and then immediately received either placebo (*n* = 34), 100/25 mg l-DOPA/carbidopa (*n* = 28), or 200/50 mg l-DOPA/carbidopa (*n* = 29). Participants completed a resting-state scan before the task and again 45 min following drug ingestion to characterize effects of l-DOPA on extinction memory neural reactivation patterns during consolidation. Twenty-four hours later, participants returned for tests of context renewal, extinction recall, and reinstatement during fMRI with concurrent skin conductance responding (SCR) assessment. Both active drug groups demonstrated increased reactivation of extinction encoding in the amygdala during the post-task resting-state scan. For SCR data, both drug groups exhibited decreased Day 2 reinstatement across all stimuli compared to placebo, and there was some evidence for decreased context renewal to the fear stimulus in the 100 mg group compared to placebo. For imaging data, both drug groups demonstrated decreased Day 2 reinstatement across stimuli in a bilateral insula network compared to placebo. There was no evidence in SCR or neural activity that l-DOPA improved extinction recall. Reactivation of extinction encodings in the amygdala during consolidation on Day 1 predicted Day 2 activation of the insula network. These results support a role for dopamine during the consolidation window in boosting reactivation of amygdala extinction encodings and reducing reinstatement, but not improving extinction recall, in women with PTSD.

## Introduction

Posttraumatic stress disorder (PTSD) is associated with marked impairment^1^. Exposure-based therapy is among the best supported interventions for PTSD^2^, yet remission rates are typically only 50–60%^3,4^. Exposure therapy is hypothesized to work via the mechanisms of fear extinction learning^5^, and considerable efforts have been made to identify ways of boosting extinction learning towards the goal of improving exposure therapy efficacy^6–8^.

The role of dopaminergic signaling during the post-fear extinction consolidation window has not been investigated in PTSD, despite a growing body of data implicating dopamine as a critical mechanism underlying fear extinction learning, consolidation, and subsequent recall^9–15^. Animal models have demonstrated that dopamine agonists delivered during or following fear extinction learning^9,12,13^ decrease subsequent fear responding. Chemogenetic studies further suggest a key role of dopaminergic neurons projecting to the striatum in mediating fear extinction learning^10,11,16^. Studies among healthy men similarly demonstrate that the dopamine precursor l-DOPA delivered following extinction learning reduced subsequent context renewal^9^ and improved extinction recall^17^, possibly by boosting extinction reactivation patterns in the ventromedial prefrontal cortex (vmPFC)^17^ or ventral tegmental area coupling with vmPFC^9^. Accordingly, boosting post-learning dopaminergic signaling is a potentially viable route towards boosting response to exposure therapy for PTSD.

However, PTSD is canonically associated with deficits in fear extinction learning, recall, and context renewal^18–20^, which might preclude the possible efficacy of manipulations that boost learning in healthy populations with intact fear extinction learning. Similarly, PTSD is associated with decreased striatal encoding of reward prediction errors^21^ and decreased cerebrospinal fluid (CSF) concentrations of dopamine metabolites during symptom provocation^22^, which might preclude the degree to which dopamine manipulations can improve fear extinction learning in PTSD patients. Thus, it is necessary to first demonstrate in a PTSD sample that boosting post-extinction dopamine signaling improves extinction learning.

This study therefore presents the first test of whether l-DOPA boosts consolidation and subsequent recall of laboratory fear extinction learning among women with PTSD. We hypothesized that l-DOPA would (1) improve context renewal (i.e., decreased fear responding in the original acquisition context), reinstatement (i.e., decrease fear responding following an unsignaled presentation of the US), and fear extinction recall (i.e., decrease fear responding in the extinction context)^23^ during the Day 2 fear-responding tests and (2) demonstrate a greater pro-extinction effect at a 100 mg dose of l-DOPA vs 200 mg due to an inverted U-shaped relationship between dopamine and learning^24–26^. We tested fear responding using skin conductance responses (SCR), large-scale neural network activation, and complementary standard voxelwise activation patterns. Finally, consistent with prior studies in healthy men^9,17^, it is also necessary to identify the acute neural mechanism through which l-DOPA improves consolidation of extinction learning in PTSD. We accordingly employed methodology^17,27,28^ to characterize neural reactivation patterns (i.e., “neural replay”^29,30^, the spontaneous reactivation of neural patterns activated during the initial memory encoding) during a resting-state scan 45 min following drug ingestion when l-DOPA would be at peak concentration to alter extinction consolidation. Consistent with prior work using animal and human models, we hypothesized l-DOPA would boost reactivation of neural patterns recruited during extinction in neurocircuitry canonically associated with fear and extinction learning and recall^31–36^ (amygdala, hippocampus, vmPFC) and within dorsal and ventral striatum, more recently implicated in fear extinction^10,11,37^.

## Materials and methods

### Methodology overview

**Fig. 1:**
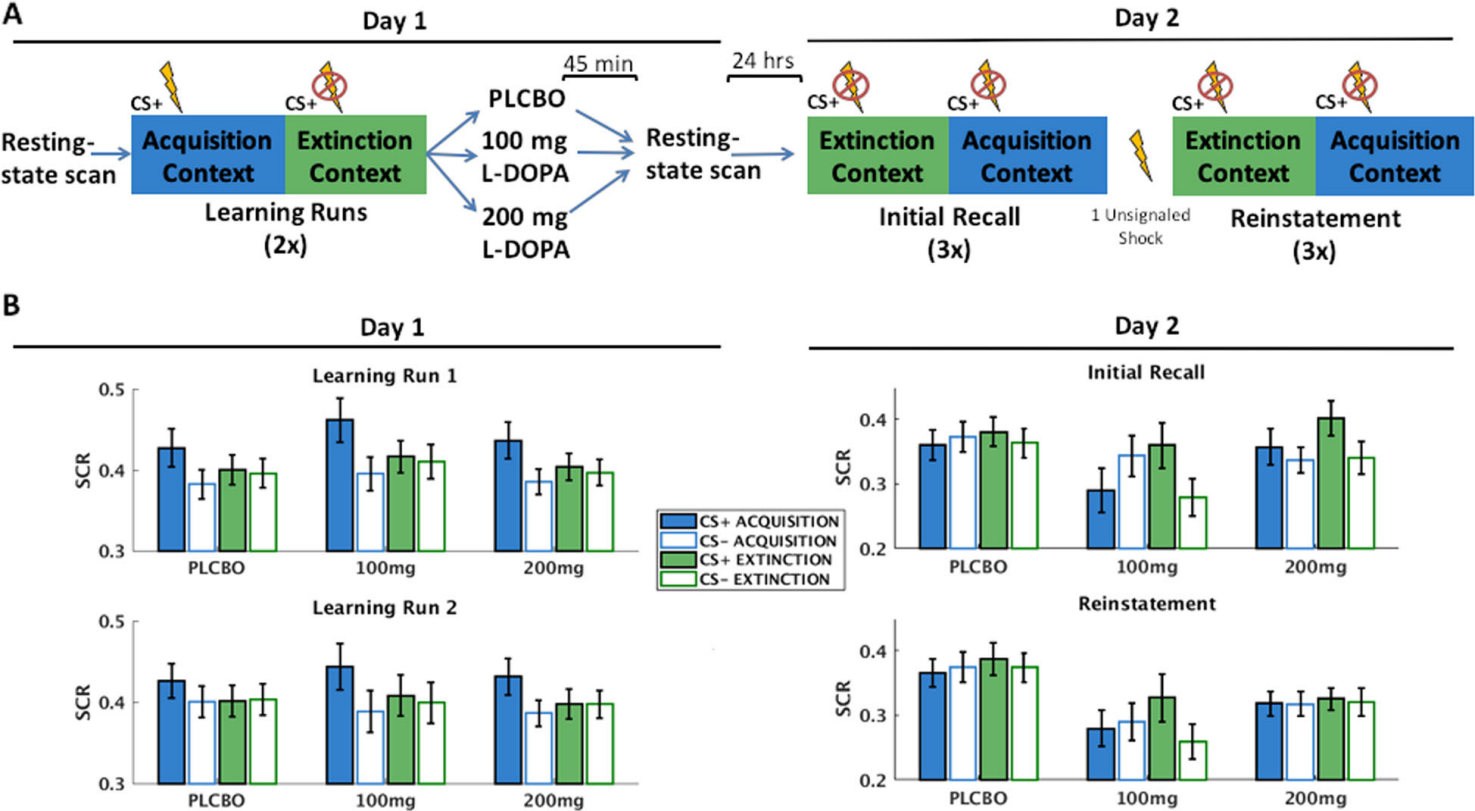
Methodology overview and skin conductance results. **a** Graphical overview of the study design. **b** Skin conductance responses (normalized) for Day 1 learning (left) and Day 2 (recall).

The overall methodology is depicted in Fig. 1a. On Day 1, participants first completed a resting-state scan, followed by a fear conditioning and extinction task. The acquisition and extinction phases of the task were conducted in distinct contexts indicated by different background colors. Immediately after completing the task, participants were removed from the scanner and received the allocated drug. Participants were then led to a quiet waiting room, with no access to phones or electronic devices and only provided with generic magazines appropriate for hospital waiting rooms. Participants had acute side effects of the drug assessed 30 min after ingestion while they were in the waiting room. Participants returned to the scanner at ~35 min after ingestion to complete positioning scans, and a resting-state scan then began exactly 45 min following drug ingestion when l-DOPA should be at peak concentration in the brain. Participants returned for Day 2 24 h later for a recall test alternating between acquisition and extinction contexts (i.e., Initial Fear Recall). A single unsignaled US was then presented (Reinstatement), followed by another recall test.

### Participants, assessments, and inclusion criteria

Inclusion criteria consisted of female sex, age 21–50 years, and current diagnosis of PTSD related to assaultive violence exposure (Table 1; Supplementary Table 1; see Supplementary Material). Ninety-one women received the allocated drug dose, and 87 of these women attended the Day 2 recall tests (see CONSORT flow chart in Supplementary Fig. 1). Participants were recruited at two different sites: University of Wisconsin Madison (UW; *n* = 48) and University of Arkansas for Medical Sciences (UAMS; *n* = 43). All procedures were approved by the UW and UAMS IRBs and all patients provided informed consent.

**Table 1:** Comparison of clinical and demographic characteristics between participant groups (placebo vs 100 mg vs 200 mg).

Variable	Placebo (a) *N* = 34	100 mg (b) *N* = 28	200 mg (c) *N* = 29	*p* values
Age (years)	33.8 (8.8)	34.8 (9.7)	34.5 (8.6)	*p*(ab) = 0.655
				*p*(ac) = 0.734
				*p*(bc) = 0.901
Education (years)	15.5 (2.9)	14.8 (2.0)	15.0 (2.6)	*p*(ab) = 0.251
				*p*(ac) = 0.457
				*p*(bc) = 0.713
Ethnicity				
White (%)	76.5	60.7	82.1	*p*(abc) = 0.168
Non-White (%)	23.5	39.3	17.9	
IQ				*p*(ab) = 0.292
Verbal	104.3 (24.0)	98.3 (19.2)	99.4 (20.0)	*p*(ac) = 0.393
				*p*(bc) = 0.839
Digit span	9.8 (2.6)	9.5 (3.3)	9.7 (1.9)	*p*(ab) = 0.695
				*p*(ac) = 0.850
				*p*(bc) = 0.799
Direct assault types (#)	6.0 (2.6)	5.6 (2.9)	5.3 (3.5)	*p*(ab) = 0.580
				*p*(ac) = 0.347
				*p*(bc) = 0.697
Sexual assault (%)	97.1	92.9	89.7	*p*(abc) = 0.493
Physical assault (%)	88.2	78.6	75.9	*p*(abc) = 0.411
Physical abuse (%)	64.7	60.7	51.7	*p*(abc) = 0.570
Age first assault (years)	8.9 (6.7)	9.4 (6.5)	9.7 (7.0)	*p*(ab) = 0.735
				*p*(ac) = 0.616
				*p*(bc) = 0.870
Age last assault (years)	28.4 (9.7)	29.3 (11.2)	27.9 (10.6)	*p*(ab) = 0.752
				*p*(ac) = 0.843
				*p*(bc) = 0.641
Time since last assault (years)	5.3 (6.7)	5.5 (7.7)	6.6 (7.8)	*p*(ab) = 0.908
				*p*(ac) = 0.491
				*p*(bc) = 0.611
Current mood disorder (%)	26.5	42.9	34.5	*p*(abc) = 0.399
Current comorbid anxiety disorder (%)	70.6	57.1	75.9	*p*(abc) = 0.294
Current GAD (%)	50.0	39.3	62.1	*p*(abc) = 0.227
CAPS-V total severity	43.8 (11.3)	40.1 (10.2)	42.3 (11.9)	*p*(ab) = 0.191
				*p*(ac) = 0.615
				*p*(bc) = 0.464
Time between first day of menstrual cycle and fear ext. paradigm (days)	23.7 (6.7)	24.4 (6.0)	25.8 (15.5)	*p*(ab) = 0.818
				*p*(ac) = 0.708
				*p*(bc) = 0.828
Birth control (%)	50.0	53.6	48.3	*p*(abc) = 0.920
Estradiol concentration^a^ (pg/mL)	1.45 (0.82)	1.43 (0.71)	1.34 (0.49)	*p*(ab) = 0.938
				*p*(bc) = 0.695
				*p*(ac) = 0.659
Daily cigarette smoker	17.6	25.0	17.2	*p*(abc) = 0.706

Note. The racial categories used by the US Census (African-American, Asian-American, Native-American, Latinx, and Pacific Islander) have been collapsed into the category “non-White”. Verbal IQ was assessed from the Receptive One-Word Picture Vocabulary Test. Digit span is from the Wechsler Adult Intelligence Scale-IV. *GAD* Generalized Anxiety Disorder, *CAPS* Clinician Administered PTSD Scale DSM-V.

^a^Estradiol concentration was calculated using enzyme immunoassay upon samples collected immediately following the second scan session. Salivary samples were only available among a subset of participants across both sites and drug groups: *N*_a_ = 21, *N*_b_ = 18, *N*_c_ = 15.

### Randomization

Participants were randomized using blocked stratified randomization (see Supplementary Material) in a double-blind manner.

### Fear conditioning, fear extinction, and fear recall task

The task used here (Supplementary Fig. 2) was modeled after a prior study among healthy adults testing the impact of l-DOPA on context renewal^9^. The unconditioned stimulus (US) was an electric shock. Conditioned stimuli consisted of triangles and circles. The US occurred 2.5 s following CS+ onset with a 50% reinforcement schedule during the acquisition phase. Colored backgrounds distinguished the acquisition and extinction contexts. No shocks occurred during the extinction phase. The task alternated between acquisition and extinction phases, with two presentations of each phase. More details are provided in the supplement.

Participants completed the Day 2 fear recall task 24 h following Day 1 Learning. The task presented two CS+ and CS− stimuli per context (acquisition and extinction contexts from Day 1) for a total of three context presentations (6 total CS presentations each), with no shock presentations. During this initial recall test (i.e., Initial Fear Recall), responding to the CSs in the extinction context reflects extinction recall, whereas responding to the CSs in the acquisition context reflects renewal. After this test, participants then received a single unsignaled US presentation to promote reinstatement before completing the recall task again (i.e., Reinstatement).

### Study medication

Participants were randomized to either placebo (*n* = 34), 100/25 mg l-DOPA/carbidopa (*n* = 28), or 200/50 mg l-DOPA/carbidopa (*n* = 29). Consistent with prior studies^9,25^, 100 mg was chosen as an optimal dose to boost learning, while 200 mg was chosen as a suprathreshold dose. Side effects were assessed at 30 min and 24 h following ingestion (Supplementary Table 2).

### Skin conductance preprocessing

Consistent with prior studies^9,38^, participants whose Day 2 SCR data showed excessive artifact or flat responding were removed from Day 2 SCR analyses (*n* = 19; *n* = 66 analyzed for Day 2). This amount of data loss (22%) is commensurate with prior fear extinction studies using SCR^9,19,38^. Further details are provided in Supplementary Material.

### Skin conductance analysis

Day 1 fear conditioning and extinction SCR data were analyzed with linear mixed-effects models (LMEMs), including factors for drug group (dummy coded with the placebo group as reference) × CS × context × slope interactions as well as additional covariates for age, education, site (UW vs UAMS), and PTSD symptom severity (CAPS total severity). The slope parameter in the LMEM is a linear regressor to explicitly account for habituation to the stimuli across the different blocks^39^. To control for degree of extinction learning on Day 1, SCR to the CS+ and CS− during the last extinction block on Day 1 were included as covariates in all Day 2 SCR analyses.

The omnibus LMEMs for Day 2 recall tests were drug group (again dummy coded) × CS × context × test phase (Initial Fear Recall vs Reinstatement) × slope, with additional covariates for day 1 SCR to the CS+ and CS− during the last extinction block, age, education, site, and PTSD symptom severity. Matlab R2016a was used for all skin conductance analyses and verification of statistical assumptions.

### Magnetic resonance imaging (MRI) and data acquisition

See Supplementary material.

### Image preprocessing

See Supplementary material.

### Independent component analysis

Given contemporary emphasis on large-scale neural networks and recent network analyses of fear learning^40–42^, primary analyses used independent component analysis (ICA) to identify large-scale networks of spatially distributed patterns of temporal coactivation^43^. A model order of 35 was used as a tradeoff between component estimation reliability and interpretability. ICA was implemented using GIFT in Matlab R2016a.

### Resting-state neural reactivation analyses

To investigate the acute mechanisms by which l-DOPA boosts consolidation of extinction learning, we followed the methodology of a recent study^17^ to define the impact of l-DOPA on neural reactivations during the resting-state task 45 min following pill ingestion. Full details are provided in the supplement. Briefly, multivariate patterns of the CS offsets (i.e., the time at which the prediction error occurs) for each stimulus type and context during the Day 1 learning task were defined for each participant in a given region-of-interest (ROI), allowing us to quantify the number of reactivations of this neural pattern during consolidation at rest. Consistent with canonical fear extinction circuitry^31,32,34^, the ROIs tested included the vmPFC, amygdala, and hippocampus. Consistent with recent data demonstrating that dopaminergic projections to the striatum mediate extinction learning^10,11,37^, we also tested separate dorsal and ventral striatum ROIs. The impact of l-DOPA on log-transformed neural reactivations^17^ were then tested with LMEMs, including factors for CS, context, and drug group (dummy coded with placebo as reference), and identical covariates as described above. Bonferroni correction controlled for alpha inflation (i.e., *p* < 0.0056). Matlfccab R2016a was used for these analyses.

### Fear conditioning, extinction, and recall imaging task network analysis

We identified 13 functional networks theoretically related to learning, dopaminergic projections, or PTSD (i.e., excluding 22 networks that represented either motion artifact, CSF, or networks of non-interest such as motor and visual cortex; Supplementary Fig. 3). We regressed each network’s timecourse onto the corresponding task design matrix (calculated with AFNI’s 3dDeconvolve) from Day 1 or Day 2 to characterize functional activation of the network (further details in supplement). For group-level analyses, beta coefficients defining functional activation for each network for each participant were then compared between groups using LMEMs, in which the beta coefficients were regressed onto the dummy-coded drug group (100 mg vs placebo and 200 mg vs placebo) × CS × context × test phase interactions as well as covariates for age, education, site, head motion, PTSD symptom severity, and degree of functional network activation during Day 1 (i.e., controlling for individual differences in activation of the network during initial learning). Bonferroni correction controlled for alpha inflation (i.e., *p* < 0.0038). Matlab R2016a was used for these analyses.

### Voxelwise activation analyses during Day 2 recall

Given previous focus on univariate analyses in the fear extinction literature^18,44–46^, we also report results from standard voxelwise general linear models that used identical design matrices as the ICA network analyses and were implemented with AFNI. Second-level voxelwise analyses used identical LMEMs as the ICA network analyses. The second-level analysis was masked with a group-level gray matter mask defined from individual subjects’ segmented anatomical scans. Cluster-level thresholding^47^ controlled for voxelwise comparisons using an uncorrected *p* < 0.001 and cluster size *k* ≥ 18. These analyses were implemented using a combination of Matlab R2016a and AFNI.

## Results

### Impact of l-DOPA on physiological measures of fear recall

*Day 1 fear acquisition and extinction learning*. Mean SCR per group per condition is indicated in Fig. 1b (left-hand portion). The LMEM demonstrated the expected CS × context interaction, *t*(9976) = 3.73, *p* < 0.001. Post hoc tests demonstrated this interaction was due to higher SCR to the CS+ vs CS− in the acquisition context, *t*(4264) = 3.64, *p* < 0.001, but not during the extinction context, *t*(5683) = 0.19, *p* = 0.85. The LMEM did not identify differences between drug groups in SCR towards any of the stimuli (all *p*s > 0.35).

*Day 2 fear recall*. Mean SCR per group per condition is indicated in Fig. 1b (right-hand portion). Full results of the LMEMs and impact of covarying for psychiatric medications and side effects are included in Supplementary Table 3. For clarity we focus here on primary results for the Initial Fear Recall and Reinstatement tests.

Replicating altered context renewal in PTSD^19^, the LMEM conducted for the Initial Fear Recall phase demonstrated across drug groups a CS × context interaction, *t*(713) = −3.61, *p* < 0.001, and post hoc tests demonstrated this interaction was due to greater SCR towards the CS+ vs CS− in the extinction context, *t*(353) = 4.44, *p* < 0.001, and no differential SCR in the acquisition context, *t*(353) = −0.83, *p* = 0.41. The LMEM additionally demonstrated a context × CS × drug group interaction, *t*(697) = −2.57, *p* = .01. Providing support for decreased context renewal in the 100 mg group, post hoc tests demonstrated this interaction was driven by lower SCR to the CS+ in the acquisition context in the 100 mg compared to both the placebo group, *t*(172) = −2.34, *p* = 0.02, and the 200 mg group, *t*(172) = −2.13, *p* = 0.035. Possibly consistent with increased discrimination between threat and safety, post hoc tests also demonstrated lower SCR to the CS− in the extinction context in the 100 mg group compared to both the placebo, *t*(172) = −2.53, *p* = 0.012, and the 200 mg group, *t*(172) = 2.08, *p* = 0.039. Post hoc tests did not provide evidence for improved fear extinction recall in either 100 or 200 mg groups (i.e., no reduced SCR to CS+ in extinction context).

Supporting decreased sensitivity to reinstatement in the active drug vs placebo groups, the omnibus LMEM demonstrated a test phase (Initial Fear Recall vs Reinstatement) × 100 mg vs placebo interaction, *t*(1370) = 2.67, *p* = 0.008, and similar trend for test phase × 200 mg vs placebo interaction, *t*(1370) = 1.84, *p* = 0.067. Post hoc tests demonstrated that the placebo group showed no change in SCR during Reinstatement compared to Initial Fear Recall; all *t*(509) < 1.19, *p*s > 0.24. By contrast, the 100 mg group demonstrated an overall reduction in SCR during Reinstatement, *t*(398) = −2.43, *p* = 0.016, suggesting less sensitivity to reinstatement. Further suggesting reduced reinstatement, post hoc tests also demonstrated lower SCR in the 100 mg compared to the placebo group towards all stimuli during Reinstatement (all *p*s < 0.049), and the 200 mg group demonstrated significantly lower SCR compared to placebo group towards all stimuli (all *p*s < 0.041) except the CS− in the acquisition context (*p* = 0.11). Supplemental analyses further isolated the reinstatement effect in the 100 mg group (Supplemental Fig. 4; see Supplementary Material).

### Impact of l-DOPA on functional network activation during fear recall tests

**Fig. 2:**
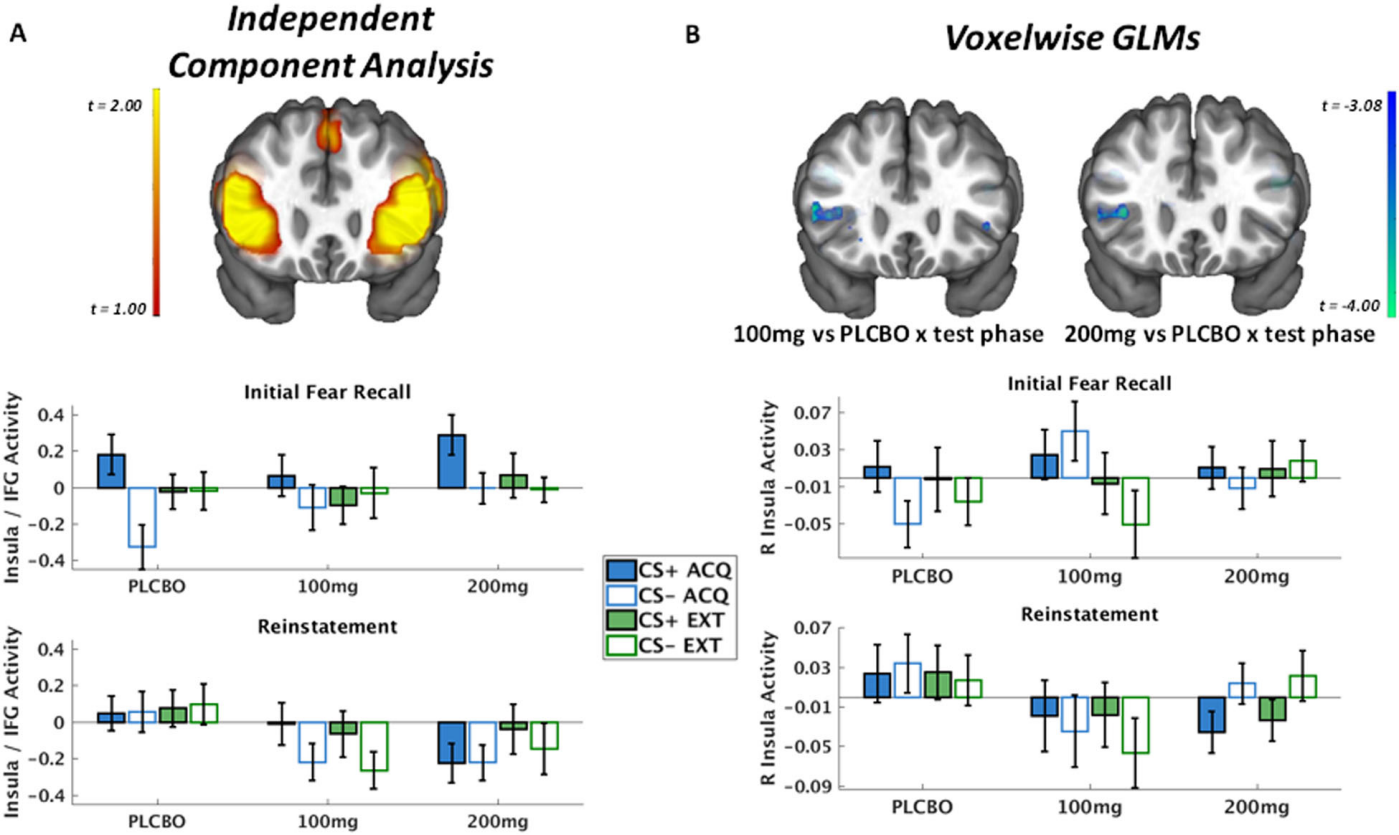
Results from Independent Component Analysis (ICA) and voxelwise imaging analyses. **a** Depiction of functional network identified with ICA with peak loadings in anterior insular cortex, inferior frontal gyri (IFG), and middle frontal gyri. Bar graphs indicate mean functional activation of the insula/IFG network across groups to each task condition separately for each test phase. **b** Corresponding depiction of a cluster in the right anterior insula identified with voxelwise GLMs corrected for whole-brain comparison where activity differed between the 100mg vs placebo groups and 200mg and placebo groups. The bar graphs indicate the corresponding mean activity in the anterior insula within an 8-mm sphere centered at *X* = 38, *Y* = 22, *Z* = 5 and chosen based on a meta-analysis of fear extinction recall among healthy human participants^45^.

Controlling for multiple comparisons, only one functional network demonstrated a significant effect with drug group—a network with peak loadings in bilateral anterior insula (AI) and inferior frontal gyri (IFG) (Fig. 2a). The omnibus LMEM for this network demonstrated a test phase × 100 mg vs placebo interaction, *t*(554) = −2.054, *p* = 0.04, and test phase × 200 mg vs placebo interaction, *t*(554) = −3.52, *p* < 0.001. Notably, this same network was robustly activated to the CS+ vs CS−, regardless of context, during Day 1, *t*(272) = 6.80, *p* < 0.001. We probed these interactions analyzing effects separately in Initial Fear Recall and Reinstatement.

All groups demonstrated comparable context renewal during Initial Fear Recall in the AI/IFG network, supported by a CS × context interaction, *t*(278) = 0.006 in the LMEM, and no interaction with drug group. Post hoc tests demonstrated this interaction was attributable to greater activity to the CS+ compared to CS− in the acquisition context, *t*(136) = 3.85, *p* < 0.001. Post hoc tests also decomposed the test phase × drug group interactions from the LMEM, and found that in the placebo group, there was a trend for overall increased activation during Reinstatement compared to Initial Fear Recall, *t*(210) = 1.61, *p* = 0.11. In the 100 mg group, the direction of the test phase effect was negative and non-significant, *t*(162) = −1.31, *p* = 0.19. In the 200 mg group, there was a significant decrease in overall activation during Reinstatement compared to Initial Fear Recall, *t*(170) = −3.79, *p* < 0.001. Similarly suggesting decreased sensitivity to reinstatement, post hoc tests also demonstrated that both drug groups exhibited overall decreased activation during the Reinstatement test compared to the placebo group, *t*s < −2.02, *p*s < 0.044, and no interaction with CS or context.

### Impact of l-DOPA on voxelwise activation during fear recall tests

**Fig. 3:**
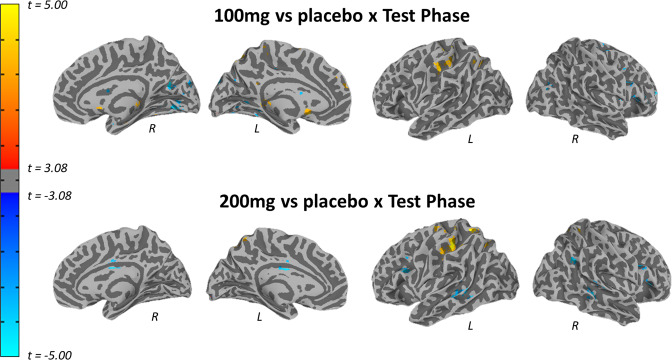
Voxelwise LME results for Day 2 100 mg vs placebo × test phase interaction (top) and 200 mg vs placebo × test phase interaction (bottom). Results are thresholded and displayed at corrected *p* < 0.05. Supplementary Table 4 provides full results of cluster sizes and coordinates.

The voxelwise LMEMs also demonstrated significant clusters (controlling for voxelwise comparisons) for the test phase × 100 mg vs placebo and test phase × 200 mg vs placebo interactions in the right anterior insula cortex (Figs. 2b and 3, Supplementary Table 4), indicating overall decreased activation during Reinstatement compared to Initial Fear Recall in the drug groups compared to placebo groups. Importantly, the placebo group demonstrated significantly increased activity in this cluster following reinstatement compared to initial recall, *t*(211) = 4.4, *p* < 0.001. To confirm the reinstatement effect in the placebo group, we also performed a voxelwise analysis just within the placebo group and again demonstrated significantly increased activation in the right anterior insula following reinstatement compared to initial fear recall (Supplementary Fig. 5). Further, the anterior insula cluster identified here appeared to overlap with the same cluster in a recent meta-analysis of fear extinction recall in healthy adults^45^. To confirm this, we conducted identical LMEMs on the mean voxel activity within an 8-mm spherical ROI centered at the right anterior insula site from the meta-analysis, *X* = 38, *Y* = 22, *Z* = 5, and similarly observed test phase × 100 mg vs placebo interactions, *t*(547) = −2.90, *p* = 0.004, and test phase × 200 mg vs placebo interactions, *t*(547) = −2.29, *p* = 0.022. Notably, the anterior insula was bilaterally robustly activated for the CS × context interaction during Day 1 learning (*p* < 0.05 corrected for whole-brain comparison; Supplementary Fig. 6, Supplementary Table 5).

### Impact of l-DOPA on neural reactivations during consolidation

**Fig. 4:**
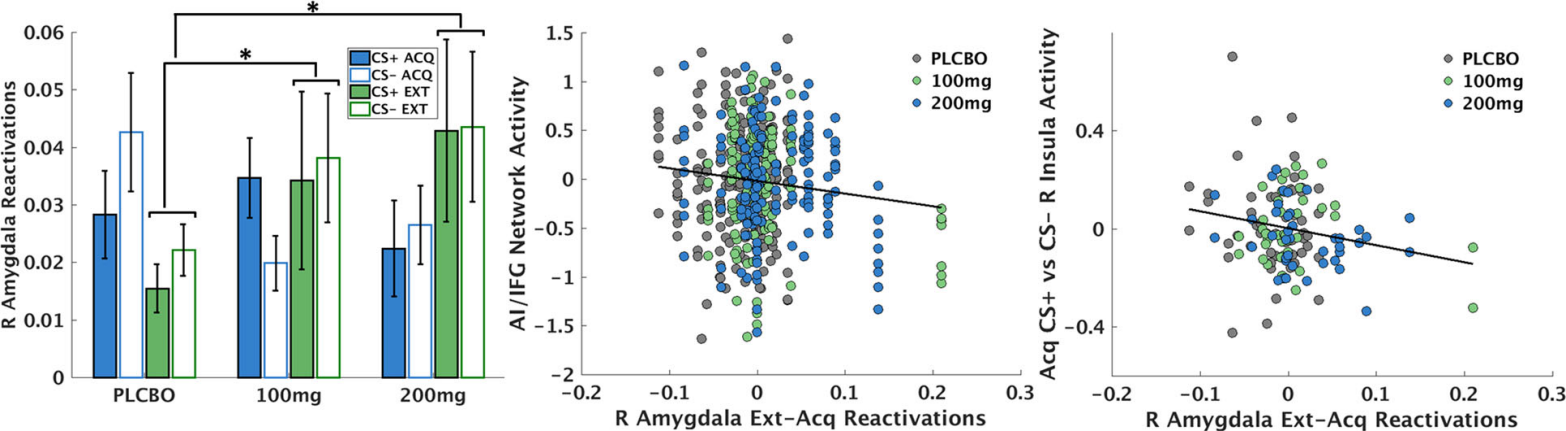
Results from neural reactivation analyses for the right amygdala. **a** l-DOPA increased the number of reactivations of extinction patterns in the right amygdala during a resting-state scan 45-min following the fear conditioning and extinction task. The *y*-axis represents the proportion of time to repetition (TRs) where a reactivation occurred. **b** Number of right amygdala extinction reactivations (compared to acquisition reactivations) during rest predicted decreased overall activation of the anterior insula/inferior frontal gyrus (AI/IFG) during the task on Day 2. **c** Number of right amygdala extinction reactivations (compared to acquisition reactivations) during rest predicted decreased responding of the right anterior insula specifically to the CS+ in the acquisition context on Day 2.

Controlling for multiple comparisons, neural reactivations in only one ROI demonstrated a significant relationship with drug group—the right amygdala. The LMEM identified both a 100 mg vs placebo × context interaction, *t*(272) = −2.11, *p* = 0.036 and a 200 mg vs placebo × context interaction, *t*(272) = −3.30, *p* = 0.001. Post hoc tests demonstrated these interactions were attributable to more amygdala reactivation of CS offsets in the extinction vs acquisition contexts in the active drug groups vs placebo group (Fig. 4a), with no difference between groups for CS+ and CS− in either context.

We then tested whether degree of amygdala extinction reactivations predicted measures of fear recall on Day 2, using parallel LMEMs where amygdala reactivations (extinction–acquisition) were included as an additional predictor. This analysis failed to identify any statistically significant relationship between amygdala extinction reactivations and Day 2 SCR. By contrast, the LMEM identified a significant negative relationship between amygdala extinction reactivations and AI/IFG network activations, which was not specific to either CS or context, *t*(468) = −3.61, *p* < 0.001 (Fig. 4b). When examining right AI activation using the identical 8 mm ROI from the meta-analysis^45^ described above, the LMEM identified a CS × context × reactivation interaction, *t*(507) = −2.23, *p* = 0.026, such that, per post hoc tests, greater amygdala reactivations during consolidation was related to less AI activity to the CS+ vs CS− in the acquisition context (Fig. 4c), *t*(251) = −2.10, *p* = 0.037.

### Ruling out confounds due to site differences and medications

As indicated in Supplementary Figs. 7 and 8, the impact of l-DOPA on fear recall outcomes were generally consistent across sites. Supplementary Tables 6–12 demonstrate that the results are not confounded by psychiatric medications or l-DOPA side effects.

## Discussion

Across both SCR, neural network, and voxelwise indices of fear recall, the most robust association with l-DOPA was decreased fear responding following reinstatement compared to placebo. For the SCR data specifically, it is first necessary to discuss limitations of these data. The SCR in these data did not provide clear evidence of fear recall in the acquisition context, such that there was no overall increase to the CS+ compared to CS−. Given this pattern, one might question whether any fear learning and subsequent extinction learning occurred for l-DOPA to then potentiate in the active drug groups. However, interpreting SCR to the CS− in a PTSD sample is not straightforward. First, recent computational modeling studies demonstrate that SCR during fear conditioning represents both the predictive value of the cue as well as uncertainty associated with the cue^48,49^. Second, and relatedly, PTSD is associated with increased fear generalization and decreased inhibition of fear to safety stimuli^20,50^. Given these two observations, one might then expect that women with PTSD would respond to the current CS- with uncertainty and fear generalization. While there was not an overall difference in CS+ vs CS− SCR in the acquisition context, there was evidence across all groups for increased CS+ vs CS− SCR in the extinction context. This altered context renewal is potentially consistent with a prior report of altered context renewal in PTSD^19^, though differences in the task used here and the prior report make direct comparisons difficult. These considerations in interpreting the current SCR data should be kept in mind when interpreting the impact of l-DOPA on fear recall.

The 100 mg group demonstrated decreased SCR to the CS+ in the acquisition context (i.e., decreased context renewal), but no differences in the extinction context (i.e., no differences in extinction recall), compared to the placebo group. This SCR pattern is potentially consistent with a prior l-DOPA study among healthy adult men that found decreased context renewal, but not fear extinction recall, compared to placebo^9^. The current findings are also consistent with decreased context renewal, but not fear extinction recall, in a rodent model where D1 receptors were specifically activated in the dorsal striatum^11^. The 100 mg group also demonstrated decreased CS− SCR in the extinction context relative to other groups, potentially suggesting increased threat-safety discrimination during fear extinction recall. These data suggest that while 100 mg of l-DOPA may weaken SCR indices of context renewal^9,11^ and improve threat-safety discrimination, it does not improve fear extinction recall. Future research is needed to clarify the specific mechanisms governing context renewal vs extinction recall to better understand how l-DOPA and dopamine might differentially contribute to these processes.

While the SCR evidence for decreased context renewal in the 100 mg vs placebo group is difficult to interpret given the overall inverted context renewal in the SCR data, the data suggesting decreased reinstatement in both l-DOPA groups was more straightforward. Following reinstatement, the placebo group maintained initial levels of SCR to the conditioned stimuli. By contrast, the l-DOPA groups demonstrated reduced SCR across stimuli following reinstatement relative to the placebo group. The reduced reinstatement was not specific to either CS or context, suggesting both reduced fear reactivation to the CS+ and reduced fear generalization to the CS−. Given that the half-life of l-DOPA is 1–2 h^51,52^, it is unlikely the drug had any direct impact on responding 24 h later. As such, a more plausible explanation would be that l-DOPA alters the consolidation of learning on Day 1 to impact subsequent responding during Day 2, but that the boosting of extinction consolidation is potentially subtle and requires a reactivating event, such as reinstatement, to unmask. Future research is needed to corroborate these findings and pinpoint the specific role of dopaminergic consolidation processes on reducing fear reinstatement rather than improving fear extinction recall.

The brain imaging data provide corroborative support for the inference of l-DOPA reducing reinstatement by suggesting increased activation in the placebo group following reinstatement, yet decreased activation following reinstatement in the 100 and 200 mg groups, in both a large-scale AI/IFG network identified with ICA as well as a cluster in the right anterior insula identified with voxelwise LMEMs. Further, while the SCR data did not provide strong evidence for fear recall to the CS+ vs CS− in the acquisition context (i.e., context renewal), the AI/IFG network demonstrated clear evidence for fear recall across groups in the first test phase, followed by overall attenuation of network activity in the active drug groups compared to placebo group following reinstatement. Further, the right anterior insula cluster from voxelwise analyses demonstrated clear evidence for fear reinstatement in the placebo group, lending stronger support for decreased fear reinstatement in this cluster in the active drug groups. Providing further support, the clusters of reduced activity in the anterior insula in the drug groups overlap with the anterior insula cluster identified as robustly engaged during fear recall tests following extinction learning among healthy adults^45^. Consistent with the SCR data, reduced reinstatement in the drug groups was not specific to either CS or context, suggesting both reduced fear reactivation and reduced fear generalization. The voxelwise LMEMs (Fig. 3, Supplementary Table 4) did not reveal any evidence of an impact of l-DOPA on traditional fear extinction neurocircuitry^31^, such as vmPFC or amygdala; however, this is consistent with recent meta-analytic findings that vmPFC and amygdala are not robustly engaged during fear extinction recall^45^. It is interesting to consider higher-order processes, other than fear or extinction recall, that reduced anterior insula activity might reflect. The anterior insula is a core node of the salience network^53,54^ and robustly engaged during tasks manipulating attention and awareness^55^. As such, reduced anterior insula activity following reinstatement in the l-DOPA compared to placebo groups might reflect less saliency in the conditioned cues and/or less attention engaged towards those cues.

With respect to potential acute mechanisms by which l-DOPA boosts consolidation of extinction learning^17^, the data demonstrated increased neural reactivation of amygdala patterns engaged in response to stimulus offsets (i.e., prediction error teaching signals) during extinction in both drug groups compared to placebo. The specificity of reactivations to the extinction, rather than acquisition, offset patterns is important, as it rules out an alternative explanation of these data: that l-DOPA impairs consolidation of acquisition memories rather than boosting consolidation of extinction memories. The non-specific boosting of reactivations to both CS+ and CS− extinction offsets is also interesting, as generalization of fear to safety signals is a feature of PTSD^20,50,56,57^. By boosting reactivation of CS− extinction memories, l-DOPA may facilitate some degree of protection from fear memory generalization. We also observed corroborating functional relationships with degree of amygdala extinction reactivation patterns, such that greater reactivations were associated with less AI/IFG network overall activation and less AI activity specifically to the CS+ in the acquisition context (i.e., decreased context renewal). These functional relationships were similar but not identical to the fear recall indices directly associated with l-DOPA dose; as such, the impact of l-DOPA on fear recall and reinstatement is not wholly explained by amygdala reactivations during consolidation. Rather, there are likely other intermediate mechanisms by which l-DOPA alters consolidation of extinction learning (e.g., D1 receptor activation in dorsal striatum^11^). While we did not replicate the prior finding of l-DOPA boosting vmPFC extinction reactivation observed among healthy men^17^, the current observation of amygdala extinction reactivation is consistent with a substantial body of data demonstrating the role of amygdala plasticity in fear extinction learning^33,35,58,59^. The lack of replication of vmPFC reactivations could be due to differences between studies in sex, PTSD diagnosis, and/or task design.

An additional consideration for the observed relationships of l-DOPA with amygdala reactivations and not vmPFC reactivations is the short timing between fear acquisition and extinction in the current task. Whereas extinction learning is traditionally viewed as new learning that competes with the acquisition memory, extinction learning that occurs in close temporal proximity to acquisition learning may also involve some degree of direct degrading of the acquisition memory mediated by depotentiation of synapses within the amygdala^60^. Accordingly, l-DOPA delivered shortly after acquisition and extinction learning in the current study may have impacted a related depotentiation process that could also be reflected in the increased amygdala reactivations. Relatedly, resting-state functional MRI (fMRI) data are collected at a relatively slow frequency (i.e., 2 s TRs) and as such neural reactivation patterns detected in resting-state fMRI data could reflect both long-term depotentiation and long-term potentiation processes in the targeted brain regions. These considerations underscore the need for additional translational research to further pinpoint mechanisms of extinction consolidation.

While the results suggest the potential for targeting dopaminergic signaling as a means of boosting extinction learning in PTSD, the current study is not without limitation. First, it is relevant to mention again that the Day 2 SCR data did not provide clear evidence for increased responding to the CS+ vs CS− in the acquisition context nor did it provide clear evidence for increased responding in the placebo group following reinstatement. While the imaging data did demonstrate increased AI/IFG network activity for the CS+ vs CS− in the acquisition context and there was clear evidence of fear reinstatement in the anterior insula cluster in the placebo group, the inconsistencies in the SCR data are noteworthy nonetheless and highlight the need for replication. Second, l-DOPA also impacts serotonergic function^61^ and further study will be necessary to specifically isolate the role of dopaminergic signaling among humans. Third, given that the current sample was selected based on interpersonal traumas, replication of the results using social stimuli is warranted. Fourth, the task used here included distinct cues indicating the different contexts. It cannot be known the degree to which results would differ without context cues signaling extinction. Fifth, subsequent studies should use 3-day designs, with acquisition and extinction separated, to more clearly define the impact on extinction consolidation specifically. Sixth, it is not clear how the results would generalize to men. Seventh, psychotropic medication use was not exclusionary for this sample. While additional analyses did not support the alternative explanation that medication usage was driving results (Supplementary Material), further investigation of the robustness of l-DOPA’s impact on consolidation of learning across diverse clinical samples (e.g., with varying degrees of medication usage, comorbidity, impairment severity, etc.) is necessary.

## Supplementary information

supplemental material
