# Supplementary material for: l-DOPA and consolidation of fear extinction learning among women with posttraumatic stress disorder
Source: Transl Psychiatry. 2020 Aug 15;10:287. doi: 10.1038/s41398-020-00975-3 (PMC7429959; doi:10.1038/s41398-020-00975-3)
Supplement: Supplementary file 1 — supplemental material [file 41398_2020_975_MOESM1_ESM.docx]

**fMRI Tasks**

**Randomization**

Participants were randomized using blocked stratified randomization, in which randomization was stratified based on age (>35 or <= 35), number of comorbid diagnoses (<3 or >=3), and whether they were currently prescribed psychotropic medication (yes or no). Randomization was successful in creating groups balanced on key demographic and clinical variables (see Table 1 and supplemental Table S1).

**Sample Size**

Sample was determined via power analysis based on effect sizes of SCR data available from a prior study among healthy humans^1^.

**Resting-State Task**

Participants underwent a 7.5min resting-state task during which they were told to stay awake, focus on a fixation cross, and let their mind wander.

**Skin conductance acquisition and processing**

At both sites, SCR data were acquired on a BIOPAC MP150 Data Acquisition System using the EDA100C module with MECMRI-TRANS cable system. Data were acquired directly into BIOPAC AcqKnowledge 4.3 software at 2000 Hz (Arkansas site) or 1000 Hz (Wisconsin site). Shocks were administered via the BIOPAC STM100C module using pre-gelled electrodes placed on the skin of the fleshy portion of the mediolateral, left lower leg, directly over the tibialis anterior. SCR recording electrodes were placed on the medial portions of the thenar and hypothenar eminences of the left hand; ground electrode was placed on the ventral surface of the left wrist. Amperage on the stimulation device was set to the maximum (50 mA) to allow the greatest range of intensity selections. Participants were told to select an intensity of a 7/10 pain scale.

Consistent with prior studies^1,2^, prior to breaking of the blind and performing any analyses, participants whose Day 2 SCR data showed excessive artifact or flat responding were removed from Day 2 SCR analyses (total n=19; n=8 from placebo, n=6 from 100mg, n=5 from 200mg). This amount of data loss (22%) is commensurate with prior fear extinction studies using SCR^1–3^. Skin conductance data underwent preprocessing consistent with contemporary recommendations ^4–8,8–10^, which included, in order, 1) a 10ms median filter, 2) unidirectional butterworth filter with .0159hz and 5hz low and high pass frequencies, and 3) downsampling to 10hz. Skin conductance responses were then estimated on a trial-by-trial basis by applying the well-validated forward convolution model of skin conductance responses within a GLM approach^6,8–11^. Resulting SCRs were normalized to each individual’s max SCR per day to account for inter-individual differences in overall magnitude of SCR responding. Reinforced CS+ trials from Day 1 Acquisition phase blocks were not included in analyses to avoid any contamination of SCR responses to the stimulus with SCR responses to the shock.

**MRI conductance acquisition and processing**

At the Arkansas site, fMRI data were acquired on a Philips Achieva 3T X-series scanner using a 32-channel headcoil. T1-weighted anatomic images were acquired with a MP-RAGE sequence (matrix = 192 × 192, 160 sagittal slices, TR/TE/FA = 7.5/3.7/9°, FOV = 256, 256, 160, final resolution = 1 × 1 × 1 mm resolution). Echo planar imaging sequences were used to collect the functional images using the following sequence parameters: TR/TE/FA = 2000 ms/30 ms/90°, FOV = 240 × 240 mm, matrix = 80 × 80, 37 axial slices (parallel to AC–PC plane to minimize OFC signal artifact), slice thickness = 2.5mm, and final resolution of 3 × 3 × 3 mm.

At the UW-Madison site, fMRI data were acquired on a GE MR750 3T scanner using an 8-channel headcoil. T1-weighted anatomic images were acquired with a MP-RAGE sequence (matrix = 256x256, 156 axial slices, TR/TE/FA = 8.2ms/3.2ms/12°, FOV = 25.6cm, final resolution = 1x1x1mm). EPI sequences used to collect the functional images used the following parameters: TR/TE/FA = 2000ms/ 25 ms/ 60, FOV = 24cm, matrix = 64 x 64, 40 sagittal slices, slice thickness = 4mm, original resolution was 4 x 3.75 x 3.75, and images were resampled to match the resolution of the UAMS data of 3x3x3mm.

Image preprocessing followed standard steps and was completed using AFNI software. In the following order, images underwent despiking, slice timing correction, deobliquing, motion correction using rigid body alignment, alignment to participant’s normalized anatomical images, spatial smoothing using a 8 mm FWHM Gaussian filter (AFNIs 3dBlurToFWHM that estimates the amount of smoothing to add to each dataset to result in the desired level of final smoothing), detrending, bandpass filtering (low frequency [.0078 Hz] for task data and low and high frequency [.01Hz - .1Hz] for resting-state data), and rescaling into percent signal change. Images were normalized using the MNI 452 template brain. We corrected for head motion related signal artifacts by using motion regressors derived from Volterra expansion, consisting of [R R^2^ R_t-1_ R^2^_t-1_], where R refers to each of the 6 motion parameters, and separate regressors for mean signal in the CSF and WM. This step was implemented directly after motion correction and normalization of the EPI images in the image preprocessing stream. Additionally, we censored TRs from the first-level GLMs based on threshold of framewise displacement (FD) > 0.4. FD refers to the sum of the absolute value of temporal differences across the 6 motion parameters; thus, a cut-off of 0.4 results in censoring TRs where the participant moved, in total across the 6 parameters, more than ~0.4 mm plus the immediately following TR (to account for delayed effects of motion artifact). Additionally, we censored isolated TRs where the preceding and following TRs were censored, and we censored entire runs if more than 50% of TRs within that run were censored. This led to the removal of 10 women from task analyses (n=4 from placebo, n=3 from 100mg, n=3 from 200mg) and 7 women (n=1 from placebo, n=1 from 100mg, n=5 from 200mg) from resting-state analyses due to missing data.

The voxelwise analyses reported in the manuscript used cluster-thresholding^12^ to correct for multiple comparisons (AFNIs 3dClustSim), in which a voxel-level uncorrected p < .001 was used with a cluster threshold of k = 18 to achieve a corrected p < .05.

**Randomization Implementation**

Blocked and stratified randomization sequences were generated using custom code in Matlab. The randomization sequence and blind was maintained by an independent pharmaceutical research center. Placebo and L-DOPA were in capsule form and identical in appearance. Pills were prepacked in bottles by the independent pharmaceutical research center, and consecutively numbered for each woman according to the randomization schedule. Both participants and research staff were blind to drug allocation. Study enrolment occurred between April 2016 and May 2018 and ended after the targeted sample size was achieved. The study protocol can accessed by contacting the corresponding author.

**Additional information on task activation design matrices.**

The design matrix was similar for Day 1 and Day 2 and included regressors for the CS and context manipulations and an additional regressor for the contingency assessment rating periods. The Day 2 model included an additional regressor for linear habituation (to control for habituation/re-extinction to the stimuli across the Day 2 task^13,14^). Day 2 was modeled with two separate design matrices to differentiate Initial Fear Recall from Reinstatement.

**Resting-State Neural Reactivation Analyses**

We followed the methodology of a recent study^15^ to define the impact of L-DOPA on neural reactivations during the resting-state task 45min following pill ingestion. It relevant to mention that the prior study found that 45min following pill ingestion was the optimal time at which to detect L-DOPA impact on neural reactivation (relative to 10min and 90min following ingestion). In this methodology, we first define the multivariate patterns of activity of the CS offsets in both the acquisition and extinction contexts. There are 5 such offset types: 1) CS+ non-reinforced offsets in the acquisition context, 2) CS+ reinforced offsets in the acquisition context, 3) CS- non-reinforced offsets in the acquisition context, 4) CS+ non-reinforced offsets in the extinction context, and 5) CS- non-reinforced offsets in the extinction context.

Our hypotheses focus on encoding of the extinction context teaching signals (i.e., negative prediction errors), consistent with the hypothesized role of dopamine in boosting extinction learning. The offset patterns in the acquisition context allow us to rule out two competing hypotheses. One alternative hypothesis is that the consolidation boosting effect of L-DOPA is non-specific and impacts the acquisition memories as much as the extinction memories. If this were the case, then a main effect of L-DOPA would be seen on encoding of all teaching signals. Another alternative hypothesis is that L-DOPA may not boost consolidation of extinction memories, but rather is inhibiting consolidation of acquisition memories. If this were the case, then we would see a specific impact of L-DOPA on *decreasing* neural reactivation patterns of the reinforced CS+ in the acquisition context.

Following the prior study, we focus on the first half of the CS offset patterns in each context repetition (e.g., first 5 CS+ reinforced offsets in the first acquisition context repetition, first 5 CS+ reinforced offsets in the second acquisition context repetition, etc). That is, once the stimulus contingencies in a given context are learned, the prediction error signal decreases. As such, the later CS offset patterns would no longer be encoding prediction errors as learning has stopped or slowed. These offsets were subsequently included in the task design matrices as additional regressors of interest, allowing us to define unique patterns of activation to these teaching signals not explained by the other task design predictors of interest (i.e., stimulus onsets).

Following the prior study, we selected ROIs from the Harvard-Oxford Atlas, thresholded at 50% tissue probability. Multivariate patterns of activity to the CS offsets were then extracted for each of the ROIs of interest: bilateral amygdala, bilateral hippocampi, bilateral ventral (nucleus accumbens) and dorsal (caudate and putamen) striatum, and vmPFC. Multivariate patterns of activity within these ROIs were then linked with resting-state data in accordance with the prior study. Specifically, first-level GLMs were conducted for each participant’s Day 1 pre- and post-learning resting-state scans, in which a delta function, convolved with a canonical HRF, modeled each resting-state TR and therefore captured each potential neural reactivation that might occur during the resting-state scan. The resting-state data did not have high-pass filtering applied to it. Multivariate patterns of activity were then extracted from each ROI at each volume of the resting-state scans (225 volumes of resting-state scans). The participant’s multivariate pattern of activity during the task was then correlated, and r-to-z transformed, with the multivariate pattern of activity during each volume of both resting-state scans. The 225 correlations from the resting-state scan before the task formed a null distribution, from which the mean and standard deviation was used to transform each correlation of the offset patterns and the postextinction resting-state patterns into a *Z*-score: *Z_i_* = (*r_i_* – *μ) / σ*. In line with the prior study and other memory reactivation studies, a threshold of *Z* > 2 was used to define resting-state activation patterns as potential spontaneous neural reactivations of the offset patterns during learning. Results were essentially identical when using an alternative threshold of *Z* > 1.5. Reactivations were summed for each participant for each offset type and log transformed to account for non-normality of the data.

The analytic approach for comparing reactivation patterns used a factorial CS (CS+ vs CS-) x context (acquisition vs extinction) x drug group LME, with covariates consistent with the other analyses. It is relevant to mention that this analytic approach allows specificity with respect to which stimulus offset patterns are being reactivated and potentially boosted by L-DOPA. However, there is actually a fifth stimulus type that does not fit within this factorial design: stimulus offset patterns to the reinforced CS+ (i.e., positive prediction error teaching signals). That is, all four other stimulus offset patterns are non-reinforced stimuli. To rule out the possibility that L-DOPA impacts reactivation of these reinforced CS+ offset patterns, we conducted comparable LMEs specifically on these patterns across each ROI. There were no significant relationships between L-DOPA dose and reactivation of the reinforced CS+ offset patterns for any region-of-interest. All code implemented analyses in this manuscript are available upon request to the first author.

**Supplemental Reinstatement Analyses of Skin Conductance Responses**

To isolate the reinstatement effect (Supplemental Figure 4), we conducted parallel analyses comparing the last blocks of the initial fear recall (i.e., the last acquisition context block [two presentations per stimulus] and last extinction context block [two presentation per stimulus] of the initial fear recall ) and the first blocks following reinstatement (i.e., the first acquisition context block [two presentations per stimulus] and first extinction context block [two presentation per stimulus]). This group x block x context x CS LME model confirmed a 100mg versus placebo x block interaction, *t*(445)=2.30, *p*=.02, attributed to an overall reduction following reinstatement in the 100mg group, *t*(125)=-2.71, *p*=.008, and no change in the placebo group, *t*(161)=.13, *p*=.90. The 200mg versus placebo group x block interaction was not statistically significant, *t*(445)=1.56, *p*=.12.

Supplemental Table 1. Birth control, estrogen levels, smoking status, and psychiatric medication usage across the drug groups.

| **Variable** | **Placebo (a)**  **N=34** | **100mg (b)**  **N=28** | **200mg (c)**  **N=29** | ***p* values** |
| --- | --- | --- | --- | --- |
| **Birth control (%)** | 50.0 | 53.6 | 48.3 | p(abc) = 0.920 |
| **Estradiol concentration* (pg/mL)** | 1.45 (.82) | 1.43 (.71) | 1.34 (.49) | p(ab) = 0.938  p(bc) = 0.695  p(ac) = 0.659 |
| **Daily cigarette smoker** | 17.6 | 25.0 | 17.2 | p(abc) = 0.706 |
| **Receiving psychotherapy (%)** | 20.9 | 15.4 | 16.5 | p(abc) = 0.891 |
| **Psychotropic medication (%)**    **Antidepressants (%)**    **SSRI (%)**    **SNRI (%)**    **NDRI (%)**  **Mood**  **stabilizer/antipsychotic (%)**    **Benzo (%)**    **Stimulants (%)**  **Anticonvulsant (%)**  **Antianxiety (%)**  **DA receptor antagonist (%)**  **DA receptor agonist (%)**    **Any psychotropic med (%)** | 17.6    26.5    2.9    11.8    5.9    14.7    11.8    17.6  2.9  2.9  8.8  58.8 | 25.0    17.9    10.7    14.3    10.7  3.6    3.6  10.7  0.0  7.1  7.1  50.0 | 27.6    27.6    10.3  10.3  13.8  10.3  13.8  17.2  13.8  20.7  6.9  62.1 | p(abc) =  0.620  0.640  0.421  0.899  0.568  0.342  0.390  0.712  0.052  0.053  0.952  0.634 |

Note. Dopaminergic (DA) receptor antagonist medications include quetiapine, risperidone, olanzapine, ziprasidone, and prochlorperazine. DA receptor agonists include bupropion, amphetamines, dextroamphetamines, and methylphenidate. All participants prescribed acute-acting psychotropic medications refrained from taking that medication on the day of the scan and at least two hours afterwards. All scans occurred between the hours of 1300-1900. Benzodiazepine medication types, including alprazolam, diazepam, and lorazepam, did not differ between drug groups, *p*(abc) = 0.527. Additionally, stimulant medication types, including dextroamphetamine/levoamphetamine, phentermine, methylphenidate, and lisdexamfetamine, did not differ between drug groups, *p*(abc) = 0.440.

*Estradiol concentration was calculated using enzyme immunoassay upon samples collected immediately following the second scan session. Salivary samples were only available among a subset of participants across both sites and drug groups; N_a_=21, N_b_=18, N_c_=15.

Supplemental Table 2. Statistics for side effect group differences (placebo versus 100mg versus 200mg) experienced 30 min following drug ingestion on Day 1 and immediately upon returning to lab on Day 2.

| **Variable** | **Placebo** n=34 | **100mg** n=28 | **200mg** n=29 | **ꭓ^2^** **values** | ***p***  **values** |
| --- | --- | --- | --- | --- | --- |
| **Side Effects (Day 1)**  Blurry vision  Constipation  Difficulty urinating  Excess salivation  Dry mouth  Sleep problems  Heart flutters  Frequent urination  Headaches  Lightheadedness, dizziness  Nausea  Sexual dysfunction  Vomiting  Irregular breathing  Feeling restless or jittery  Muscle stiffness  Shaking or muscle trembling  Slowness, lethargy  Anxiety  Difficulty concentrating  Low energy, fatigue  Low/no motivation  Mood change  Aggressiveness  Memory problems | 2.9%  0.0%  0.0%  0.0%  0.0%  0.0%  0.0%  0.0%  0.0%  2.9%  5.9%  0.0%  0.0%  2.9%  **0.0%**  0.0%  0.0%  5.9%  0.0%  5.9%  2.9%  2.9%  0.0%  0.0%  0.0% | 0.0%  0.0%  0.0%  3.7%  3.7%  0.0%  3.7%  3.7%  7.4%  11.1%  3.7%  0.0%  0.0%  0.0%  **0.0%**  0.0%  3.7%  11.1%  3.7%  3.7%  7.4%  3.7%  3.7%  0.0%  0.0% | 6.9%  0.0%  0.0%  10.3%  6.9%  0.0%  0.0%  0.0%  0.0%  10.3%  17.2%  0.0%  3.4%  0.0%  **10.3%**  0.0%  3.4%  3.4%  3.4%  0.0%  6.9%  0.0%  6.9%  0.0%  3.4% | 2.090  -------  -------  3.994  2.327  -------  2.360  2.360  4.773  1.794  3.774  -------  2.127  1.666  **6.528**  -------  1.246  1.374  1.246  1.697  .719  1.013  2.327  -------  2.127 | .352  -------  -------  .136  .312  -------  .307  .307  .092  .408  .152  -------  .345  .435  **.038**  -------  .536  .503  .536  .428  .698  .603  .312  -------  .345 |
| **Side Effects (Day 2)**  Blurry vision  Constipation  Difficulty urinating  Excess salivation  Dry mouth  Sleep problems  Heart flutters  Frequent urination  Headaches  Lightheadedness, dizziness  Nausea  Sexual dysfunction  Vomiting  Irregular breathing  Feeling restless or jittery  Muscle stiffness  Shaking or muscle  Slowness, lethargy  Anxiety  Difficulty concentrating  Low energy, fatigue  Low/no motivation  Mood change  Aggressiveness  Memory problems | 3.0%  3.0%  0.0%  0.0%  6.1%  6.1%  0.0%  0.0%  15.2%  6.1%  **15.2%**  0.0%  **0.0%**  0.0%  3.0%  12.1%  **0.0%**  6.1%  6.1%  3.0%  12.1%  3.0%  12.1%  3.0%  0.0% | 0.0%  3.7%  3.7%  3.6%  7.4%  11.1%  0.0%  14.8%  18.5%  18.5%  **7.4%**  0.0%  **0.0%**  0.0%  0.0%  3.7%  **0.0%**  7.4%  0.0%  7.4%  3.7%  0.0%  11.1%  0.0%  3.7% | 11.5%  0.0%  3.8%  7.7%  7.7%  11.5%  0.0%  7.7%  15.4%  15.4%  **34.6%**  0.0%  **19.2%**  7.7%  7.7%  3.8%  **11.5%**  15.4%  3.8%  3.8%  19.2%  0.0%  11.5%  0.0%  7.7% | 4.294  .917  1.276  2.561  .071  .670  -------  5.051  .145  2.291  **6.897**  -------  **12.251**  4.725  2.361  2.184  **7.173**  1.666  1.634  .696  3.120  1.625  .015  1.625  2.561 | .117  .632  .528  .278  .965  .715  -------  .080  .930  .318  **.032**  -------  **.002**  .094  .307  .336  .**028**  .435  .442  .706  .210  .444  .993  .444  .278 |

Note. Bolded values were significantly different (*p*<.05) between groups.

Supplemental Table 3. Impact of 100mg and 200mg L-DOPA on SCR responding when considering possible confounding effect of participants taking psychotropic medications.

| Effect | Without accounting for medications | Covarying for dopamine antagonist | Removing participants taking dopamine antagonist | Covarying for dopamine agonist | Removing participants taking dopamine agonist | Covarying for any psychiatric medication use | Covarying for number of psychiatric medications |
| --- | --- | --- | --- | --- | --- | --- | --- |
| 100mg vs PLCBO | *t*(1370)=-2.92, *p* = .004 | *t*(1369)=-2.94, *p* = .003 | *t*(1286)=-2.66, *p* = .008 | *t*(1369)=-3.02, *p* = .003 | *t*(1212)=-2.88, *p* = .004 | *t*(1369)=-2.67, *p* = .008 | *t*(1369)=-3.00, *p* = .003 |
| 200mg vs PLCBO | *t*(1370)=-0.94, *p* = .35 | *t*(1369)=-0.99, *p* = .32 | *t*(1286)=-1.16, *p* = .25 | *t*(1369)=-1.01, *p* = .31 | *t*(1212)=-1.23, *p* = .22 | *t*(1369)=-0.58, *p* = .56 | *t*(1369)=-0.87, *p* = .38 |
|  |  |  |  |  |  |  |  |
| CS x 100mg vs PLCBO | *t*(1370)=1.97, *p* = .049 | *t*(1369)=1.97, *p* = .049 | *t*(1286)=2.44, *p* = .01 | *t*(1369)=1.97, *p* = .049 | *t*(1212)=1.84, *p* = .065 | *t*(1369)=1.97, *p* = .049 | *t*(1369)=1.97, *p* = .049 |
| CS x 200mg vs PLCBO | *t*(1370)=1.62, *p* = .10 | *t*(1369)=1.62, *p* = .10 | *t*(1286)=1.79, *p* = .07 | *t*(1369)=1.62, *p* = .10 | *t*(1212)=1.10, *p* = .27 | *t*(1369)=1.62, *p* = .10 | *t*(1369)=1.62, *p* = .10 |
|  |  |  |  |  |  |  |  |
| test x 100mg vs PLCBO | *t*(1370)= 2.66, *p* = .008 | *t*(1369)=2.66, *p* = .008 | *t*(1286)=3.15, *p* = .002 | *t*(1369)=2.66, *p* = .008 | *t*(1212)=2.65, *p* = .008 | *t*(1369)=2.66, *p* = .008 | *t*(1369)= 2.66, *p* = .008 |
| test x 200mg vs PLCBO | *t*(1370)= 1.84, *p* = .067 | *t*(1369)=1.83, *p* = .067 | *t*(1286)=1.66, *p* = .10 | *t*(1369)=1.84, *p* = .065 | *t*(1212)=1.83, *p* = .067 | *t*(1369)=1.87, *p* = .062 | *t*(1369)= 1.84, *p* = .066 |
|  |  |  |  |  |  |  |  |
| CS x context x 100mg vs PLCBO | *t*(1370)= -3.24, *p* = .001 | *t*(1369)=-3.24, *p* = .001 | *t*(1286)=-3.33, *p* = 0.001 | *t*(1369)=-3.24, *p* = .001 | *t*(1212)=-2.69, *p* = .007 | *t*(1369)=-3.24, *p* = .001 | *t*(1369)= -3.24, *p* = .001 |
| CS x context x 200mg vs PLCBO | *t*(1370)= -0.10, *p* = .92 | *t*(1369)=-0.10, *p* = .92 | *t*(1286)=0.07, *p* = .95 | *t*(1369)=-0.10, *p* = .92 | *t*(1212)=0.03, *p* = .98 | *t*(1369)=-0.10, *p* = .92 | *t*(1369)= -0.10, *p* = .92 |
|  |  |  |  |  |  |  |  |
| CS x test x 100mg vs PLCBO | *t*(1370)=0.13, *p* = .89 | *t*(1369)=0.13, *p* = .89 | *t*(1286)=0.17, *p* = .86 | *t*(1369)=0.13, *p* = .89 | *t*(1212)=-0.20, *p* = .84 | *t*(1369)=0.13, *p* = .90 | *t*(1369)=0.13, *p* = .89 |
| CS x test x 200mg vs PLCBO | *t*(1370)= 1.94, *p* = .053 | *t*(1369)=1.94, *p* = .053 | *t*(1286)=2.05, *p* = .041 | *t*(1369)=1.94, *p* = .053 | *t*(1212)=1.66, *p* = .097 | *t*(1369)=1.94, *p* = .053 | *t*(1369)= 1.94, *p* = .053 |
|  |  |  |  |  |  |  |  |

Note. PLCBO = placebo. CS = conditioned stimulus. Test = Fear recall test 1 (spontaneous recovery) vs fear recall test 2 (reinstatement). Context = Acquisition vs Extinction contexts. All models included additional covariates for Day1 CS+ and CS- responding from the last extinction phase, slope of SCR (i.e., linear effect of repeated stimulus presentation), site, age, ethnicity, education, and PTSD symptom severity.

| **Contrast** | **Region** | **MNI center-of-mass coordinates** | | | **Peak *t*** | **Cluster size** |
| --- | --- | --- | --- | --- | --- | --- |
|  |  | **X** | **Y** | **Z** |  |  |
| Test Phase x 100mg vs Placebo | Lingual gyrus | -10.2 | 71.3 | -0.4 | -4.84 | 53 |
|  | Left somatosensory | 39.1 | 33.3 | 55.4 | 5.09 | 43 |
|  | Right anterior insula | -47.8 | -24.4 | 8.4 | -4.97 | 42 |
|  | Left parietal | 25.8 | 62.1 | 50.5 | 4.89 | 39 |
|  | Left motor | 46.6 | 21.6 | 42.6 | 4.17 | 33 |
|  | Right middle frontal gyrus | -30.3 | -55.3 | 14.6 | -4.76 | 32 |
|  | Right middle frontal gyrus | -45.9 | -9.3 | 30.2 | -4.73 | 32 |
|  | Cuneus | -14.6 | 87.5 | 18.7 | -6.14 | 25 |
|  | Cerebellum | -3.1 | 56.6 | -17.6 | 4.47 | 22 |
|  | Right putamen | -31.0 | -9.6 | 7.4 | -3.86 | 19 |
|  | Right parietal | -41.0 | 75.9 | 20.5 | -4.24 | 19 |
|  | Cuneus | -18.8 | 64.8 | 20.3 | -4.99 | 18 |
|  | Left premotor | 26.3 | 21.3 | 63.8 | 5.25 | 18 |
|  | Right premotor | -28.6 | 11.4 | 65.2 | -5.00 | 18 |
| Test Phase x 200mg vs Placebo | Left somatosensory cortex | 43.2 | 31.7 | 46.6 | 5.70 | 90 |
|  | Left parietal cortex | 21.4 | 54.6 | 62.2 | 5.49 | 68 |
|  | Right superior temporal sulcus | -57.1 | 41.5 | 0.3 | -4.66 | 57 |
|  | Left superior temporal sulcus | 58.7 | 40.6 | 1.9 | -4.25 | 47 |
|  | Thalamus | -9.9 | 10.7 | 20.4 | -4.86 | 37 |
|  | Left middle frontal gyrus | 47.8 | -14.0 | 24.8 | -5.70 | 34 |
|  | Precuneus | 18.7 | 64.6 | 49.2 | 4.42 | 33 |
|  | Right anterior insula | -44.9 | -21.7 | 8.0 | -4.79 | 29 |
|  | Right parietal cortex | -42.1 | 58.3 | 32.5 | -4.92 | 24 |
|  | Right parietal cortex | -18.3 | 52.6 | 61.0 | 5.42 | 23 |
|  | Left premotor | 24.9 | 17.0 | 65.4 | 5.02 | 23 |
|  | Right middle frontal gyrus | -46.6 | -10.3 | 28.2 | -4.17 | 21 |
|  | Left motor cortex | 33.9 | 31.8 | 65.4 | 4.68 | 18 |

Supplemental Table 4. Significant clusters of activation (p < .05 corrected for multiple comparisons) for the test phase (Fear Recall Test 1 vs Fear Recall Test 2) x drug group (placebo vs 100mg vs 200mg) interaction from the linear mixed-effects analysis on Day 2 Recall imaging data.

Supplemental Table 5. Significant clusters of activation (p < .05 corrected for multiple comparisons) for the CS (CS+ vs CS-) x context (Acquisition vs Extinction) interaction from the linear mixed-effects analysis on Day 1 Learning imaging data.

| **Region** | **MNI center-of-mass coordinates** | | | **Peak *t*** | **Cluster size** |
| --- | --- | --- | --- | --- | --- |
|  | **X** | **Y** | **Z** |  |  |
| Pre-SMA | -1.8 | 21.7 | 57.7 | 6.25 | 780 |
| Left pre-motor | 42.7 | 10.3 | 47.5 | 6.59 | 164 |
| Left posterior insula | -52.9 | 35.3 | 19.8 | 5.10 | 157 |
| Right pre-motor | -43.0 | 11.8 | 48.2 | 6.35 | 156 |
| Left anterior insula | 31.8 | -14.2 | 6.1 | 5.28 | 123 |
| Right posterior insula | 49.6 | 31.8 | 19.0 | 4.83 | 99 |
| Left inferior frontal gyrus | 54.1 | -3.2 | 12.2 | 5.68 | 84 |
| Thalamus | 0.0 | 20.0 | 4.1 | 5.32 | 77 |
| Left parietal cortex | 44.0 | 65.6 | 34.9 | -4.49 | 73 |
| Right parietal cortex | -46.3 | 62.3 | 33.7 | -4.05 | 60 |
| right inferior frontal gyrus | -52.1 | 0.1 | 9.1 | 5.05 | 52 |
| Right anterior insula | -33.4 | -18.0 | 10.1 | 5.59 | 46 |
| Right superior frontal gyrus | -41.6 | -16.7 | 46.2 | -5.04 | 45 |
| Lingual gyrus | -20.3 | 62.5 | 6.5 | 4.56 | 37 |
| Right middle frontal gyrus | -38.5 | -50.1 | 11.6 | -4.55 | 32 |
| Dorsal anterior cingulate cortex | 2.9 | -25.8 | 29.6 | 3.96 | 32 |
| Posterior cingulate cortex | -0.7 | 65.6 | 33.2 | -4.41 | 32 |
| Left superior frontal gyrus | 38.8 | -14.2 | 47.6 | -4.07 | 32 |
| Right superior frontal gyrus | -15.9 | -38.6 | 49.2 | -4.23 | 27 |
| Left superior frontal gyrus | 18.3 | -25.5 | 56.2 | -4.19 | 27 |
| Right superior frontal gyrus | -15.3 | -23.4 | 60.4 | -3.89 | 23 |
| Lingual gyrus | 17.0 | 71.5 | 3.0 | 3.89 | 22 |
| Right putamen | -22.6 | -5.1 | -2.2 | 3.99 | 20 |
| Left caudate | 9.7 | -13.0 | 6.0 | 4.51 | 20 |
| Right posterior insula | -32.1 | 25.0 | 16.0 | 4.67 | 19 |

Supplemental Table 6. Impact of 100mg and 200mg L-DOPA on anterior insula region-of-interest activation when considering possible confounding effect of participants taking psychotropic medications.

| Effect | Without accounting for medications | Covarying for dopamine antagonist | Removing participants taking dopamine antagonist | Covarying for dopamine agonist | Removing participants taking dopamine agonist | Covarying for any psychiatric medication use | Covarying for number of psychiatric medications |
| --- | --- | --- | --- | --- | --- | --- | --- |
| Test x 100mg vs PLCBO | *t*(547)=-2.90, *p* = .004 | *t*(546)=-2.90, *p* = .004 | *t*(547)=-3.02, *p* = .003 | *t*(546)=-2.87, *p* = .004 | *t*(491)=-3.49, *p* < .001 | *t*(546)=-2.89, *p* = .004 | *t*(546)=-2.88, *p* = .004 |
| Test x 200mg vs PLCBO | *t*(547)=-2.29, *p* = .023 | *t*(546)=-2.29, *p* = .023 | *t*(547)=-2.14, *p* = .033 | *t*(546)=-2.30, *p* = .022 | *t*(491)=-2.50, *p* = .013 | *t*(546)=-2.26, *p* = .024 | *t*(546)=-2.28, *p* = .023 |
|  |  |  |  |  |  |  |  |

Note. PLCBO = placebo. Test = Fear recall test 1 (spontaneous recovery) vs fear recall test 2 (reinstatement). All models included additional covariates for slope of responding (i.e., linear effect of repeated stimulus presentation), site, age, ethnicity, education, and PTSD symptom severity.

Supplemental Table 7. Impact of 100mg and 200mg L-DOPA on anterior insula / inferior frontal gyrus network activation when considering possible confounding effect of participants taking psychotropic medications.

| Effect | Without accounting for medications | Covarying for dopamine antagonist | Removing participants taking dopamine antagonist | Covarying for dopamine agonist | Removing participants taking dopamine agonist | Covarying for any psychiatric medication use | Covarying for number of psychiatric medications |
| --- | --- | --- | --- | --- | --- | --- | --- |
| Test x 100mg vs PLCBO | *t*(554)=-2.05, *p* = .04 | *t*(553)=-2.06, *p* = .04 | *t*(538)=-2.27, *p* = .024 | *t*(554)=-2.05, *p* = .04 | *t*(498)=-2.38, *p* = .018 | *t*(553)=-2.08, *p* = .038 | *t*(553)=-2.05, *p* = .04 |
| Test x 200mg vs PLCBO | *t*(554)=-3.53, *p* < .001 | *t*(553)=-3.53, *p* < .001 | *t*(538)=-3.36, *p* < .001 | *t*(554)=-3.53, *p* < .001 | *t*(498)=-4.13, *p* < .001 | *t*(553)=-3.53, *p* < .001 | *t*(554)=-3.52, *p* < .001 |
|  |  |  |  |  |  |  |  |

Note. PLCBO = placebo. Test = Fear recall test 1 (spontaneous recovery) vs fear recall test 2 (reinstatement). All models included additional covariates for slope of responding (i.e., linear effect of repeated stimulus presentation), site, age, ethnicity, education, and PTSD symptom severity.

Supplemental Table 8. Impact of 100mg and 200mg L-DOPA on amygdala reactivation patterns during resting-state FC 45min after drug ingestion when considering possible confounding effect of participants taking psychotropic medications.

| Effect | Without accounting for medications | Covarying for dopamine antagonist | Removing participants taking dopamine antagonist | Covarying for dopamine agonist | Removing participants taking dopamine agonist | Covarying for any psychiatric medication use | Covarying for number of psychiatric medications |
| --- | --- | --- | --- | --- | --- | --- | --- |
| Context x 100mg vs PLCBO | *t*(272)=-2.11, *p* = .036 | *t*(271)=-2.11, *p* = .036 | *t*(264)=-2.25, *p* = .025 | *t*(271)=-2.11, *p* = .036 | *t*(244)=-1.92, *p* = .055 | *t*(271)=-2.11, *p* = .036 | *t*(271)=-2.11, *p* = .036 |
| Context x 200mg vs PLCBO | *t*(272)=-3.30, *p* = .001 | *t*(271)=-3.30, *p* = .001 | *t*(264)=-3.38, *p* < .001 | *t*(271)=-3.30, *p* = .001 | *t*(244)=-2.36, *p* = .019 | *t*(271)=-3.30, *p* = .001 | *t*(271)=-3.30, *p* = .001 |

Note. PLCBO = placebo. All models included additional covariates for baseline resting-state FC, site, age, ethnicity, education, and PTSD symptom severity.

Supplemental Table 9. Impact of 100mg and 200mg L-DOPA on SCR responding when considering possible confounding effect of L-DOPA side effects.

| Effect | Without accounting for side effects | Covarying for Day 1 and Day 2 Side Effects |
| --- | --- | --- |
| 100mg vs PLCBO | *t*(1370)=-2.92, *p* = .004 | *t*(1355)=-2.72, *p* = .007 |
| 200mg vs PLCBO | *t*(1370)=-0.94, *p* = .35 | *t*(1355)=-1.15, *p* = .25 |
|  |  |  |
| CS x 100mg vs PLCBO | *t*(1370)=1.97, *p* = .049 | *t*(1355)=2.21, *p* = .027 |
| CS x 200mg vs PLCBO | *t*(1370)=1.62, *p* = .10 | *t*(1355)=1.63, *p* = .10 |
|  |  |  |
| test x 100mg vs PLCBO | *t*(1370)= 2.66, *p* = .008 | *t*(1355)=2.27, *p* = .02 |
| test x 200mg vs PLCBO | *t*(1370)= 1.84, *p* = .067 | *t*(1355)=1.86, *p* = .063 |
|  |  |  |
| CS x context x 100mg vs PLCBO | *t*(1370)= -3.24, *p* = .001 | *t*(1355)=-3.20, *p* = .001 |
| CS x context x 200mg vs PLCBO | *t*(1370)= -0.10, *p* = .92 | *t*(1355)=-0.10, *p* = .92 |
|  |  |  |
| CS x test x 100mg vs PLCBO | *t*(1370)=0.13, *p* = .89 | *t*(1355)=0.22, *p* = .82 |
| CS x test x 200mg vs PLCBO | *t*(1370)= 1.94, *p* = .053 | *t*(1355)=1.94, *p* = .052 |
|  |  |  |

Note. PLCBO = placebo. CS = conditioned stimulus. Test = Fear recall test 1 (initial fear recall) vs fear recall test 2 (reinstatement). Context = Acquisition vs Extinction contexts. Side effects were included in the model as a linear covariate and calculated based on the sum of the total number of side effects endorsed at 30min (Day 1) and 24hrs (Day 2) after pill ingestion. All models included additional covariates for Day1 CS+ and CS- responding from the last extinction phase, slope of SCR (i.e., linear effect of repeated stimulus presentation), site, age, ethnicity, education, and PTSD symptom severity.

Supplemental Table 10. Impact of 100mg and 200mg L-DOPA on anterior insula region-of-interest activation when considering possible confounding effect of L-DOPA side effects.

| Effect | Without accounting for side effects | Covarying for Day 1 and Day 2 Side Effects |
| --- | --- | --- |
| Test x 100mg vs PLCBO | *t*(547)=-2.90, *p* = .004 | *t*(533)=-2.85, *p* = .005 |
| Test x 200mg vs PLCBO | *t*(547)=-2.29, *p* = .023 | *t*(533)=-2.42, *p* = .016 |
|  |  |  |

Note. PLCBO = placebo. Test = Fear recall test 1 (spontaneous recovery) vs fear recall test 2 (reinstatement). Side effects were included in the model as a linear covariate and calculated based on the sum of the total number of side effects endorsed at 30min (Day 1) and 24hrs (Day 2) after pill ingestion. All models included additional covariates for slope of responding (i.e., linear effect of repeated stimulus presentation), site, age, ethnicity, education, and PTSD symptom severity.

Supplemental Table 11. Impact of 100mg and 200mg L-DOPA on anterior insula / inferior frontal gyrus network activation when considering possible confounding effect of L-DOPA side effects.

| Effect | Without accounting for side effects | Covarying for Day1 and Day2 side effects |
| --- | --- | --- |
| Test x 100mg vs PLCBO | *t*(554)=-2.05, *p* = .04 | *t*(544)=-2.19, *p* = .03 |
| Test x 200mg vs PLCBO | *t*(554)=-3.53, *p* < .001 | *t*(544)=-3.79, *p* < .001 |
|  |  |  |

Note. PLCBO = placebo. Test = Fear recall test 1 (spontaneous recovery) vs fear recall test 2 (reinstatement). Side effects were included in the model as a linear covariate and calculated based on the sum of the total number of side effects endorsed at 30min (Day 1) and 24hrs (Day 2) after pill ingestion. All models included additional covariates for slope of responding (i.e., linear effect of repeated stimulus presentation), site, age, ethnicity, education, and PTSD symptom severity.

Supplemental Table 12. Linear effect of drug group on resting-state neural reactivations 45min after drug ingestion when accounting for L-DOPA side effects.

| Effect | Without accounting for side effects | Covarying for Day 1 side effects |
| --- | --- | --- |
| Context x 100mg vs PLCBO | *t*(272)=-2.11, *p* = .036 | *t*(271)=-2.11, *p* = .036 |
| Context x 200mg vs PLCBO | *t*(272)=-3.30, *p* = .001 | *t*(271)=-3.30, *p* = .001 |

Note. Side effects were included in the model as a linear covariate and calculated based on the sum of the total number of side effects endorsed at 30min after pill ingestion. All models included additional covariates for site, age, ethnicity, education, and PTSD symptom severity.

**Supplemental Figure 1.** Participant enrollment through the study design


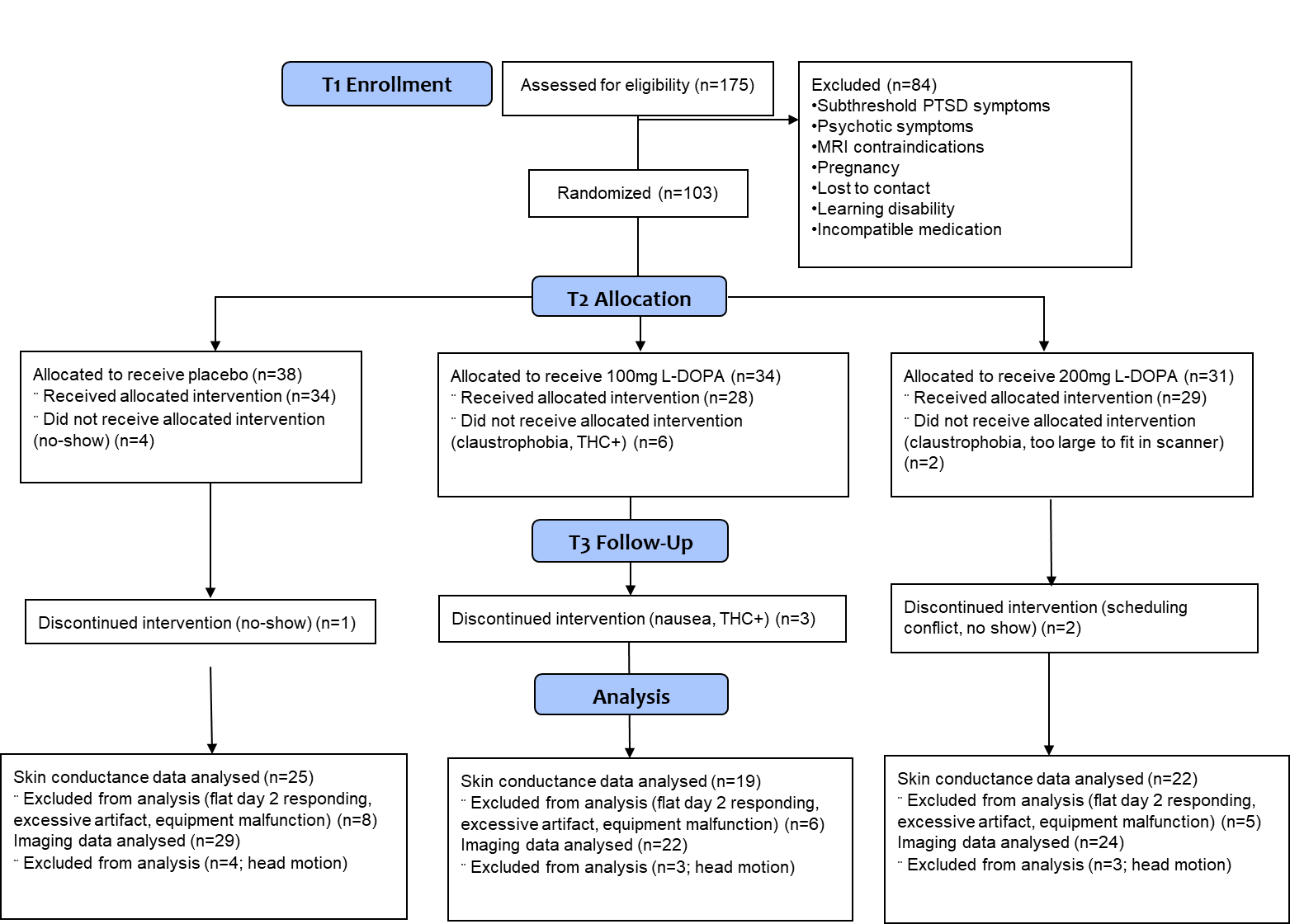


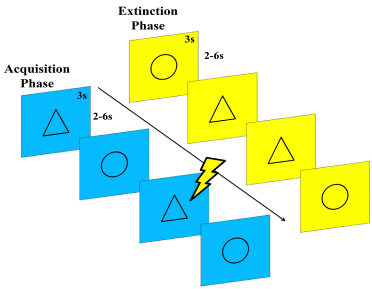


**Supplemental Figure 2.** Fear Conditioning, Fear Extinction, and Fear Recall Task Structure. On Day 1 of scanning, participants completed the fear conditioning and fear extinction task in fMRI. The unconditioned stimulus (US) was an electric shock, which participants calibrated to an intensity level of 7/10 on a Likert scale. Conditioned stimuli consisted of triangles and circles, each displayed for 3s with a jittered inter-trial interval of 2-6s, and counterbalanced across participants. An initial baseline phase consisted of 6 presentations of each stimulus with no UCS onsets. The task then alternated between acquisition and extinction phases for 156 trials, with two presentations of each phase. The acquisition phase presented each CS 18 times, with a shock occurring 2.5s following CS+ presentation with a 50% reinforcement schedule. The extinction phase presented each stimulus 18 times and no shocks occurred. Participants returned for a recall test 24 hours later using a similar procedure and task design. Participants first recalibrated the US intensity again to a 7 on a 10 point Likert scale. The recall task alternated between acquisition and extinction contexts, in a pseudorandom order for a total of 6 context repetitions, and presented two CS+ and CS- stimuli per context presentation (i.e., 12 total CS+ and CS- repetitions). Each stimulus was again presented for 3s with a jittered inter-trial interval of 2-6s. After the first run of the task, participants then received a single uncued US presentation (i.e., reinstatement), and then participants completed again the identical task as implemented in the first run.

**Supplemental Figure 3.** Networks of interest from the Independent Component Analysis (ICA) conducted on Day 1 and Day 2 fear conditioning, extinction, and recall task data. The ICA used a model order of 35, and 13 of these components were selected as networks of interest after removing components attributed to artifact (e.g., CSF, head motion) and networks of non-interest (e.g., visual and motor networks). FCT = fear conditioning tasks.


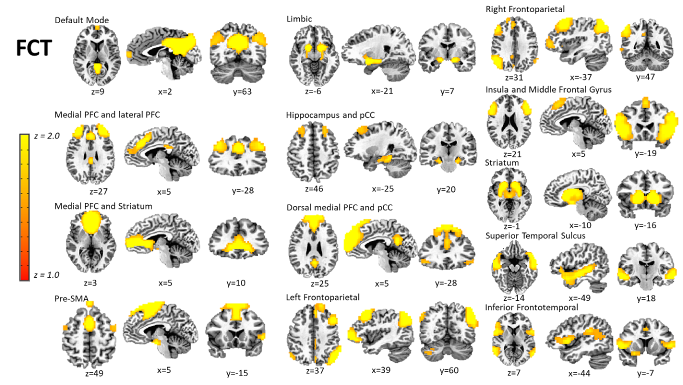


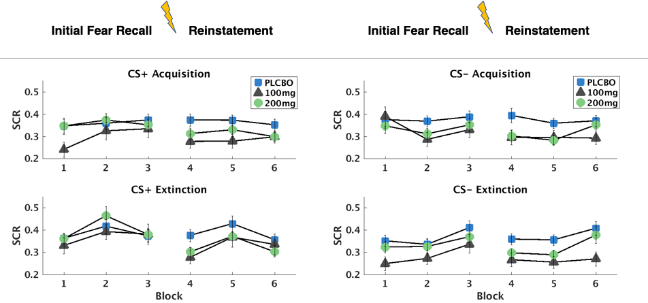


**Supplemental Figure 4.** Plots of block-by-block SCR responding to each stimulus. Each stimulus was presented twice per block. The first three blocks occur during the Initial Fear Recall test. The last three blocks occur following reinstatement. Notable is the decrease in SCR in the 100mg group, and to a lesser degree in the 200mg group, following reinstatement compared to the end of the Initial Fear Recall test.


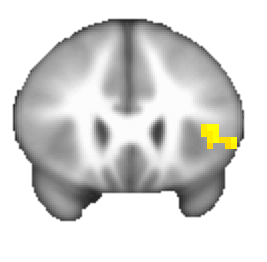


y=24


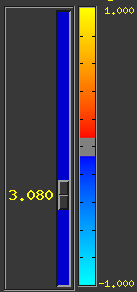


*t = 4.00*

*t = -4.00*

*t = 3.08*

*t = -3.08*

**Supplemental Figure 5.** Voxelwise LME results for Day 2 just among the placebo group, demonstrating a significant increase in responding in the right anterior insula following reinstatement. Results are thresholded at whole-brain corrected p < .05.

**Supplemental Figure 6.** Voxelwise LME results for Day 1 conditioned stimulus (CS+ vx CS-) x context (Acquisition vs Extinction) interaction. Results are thresholded at whole-brain corrected p < .05.


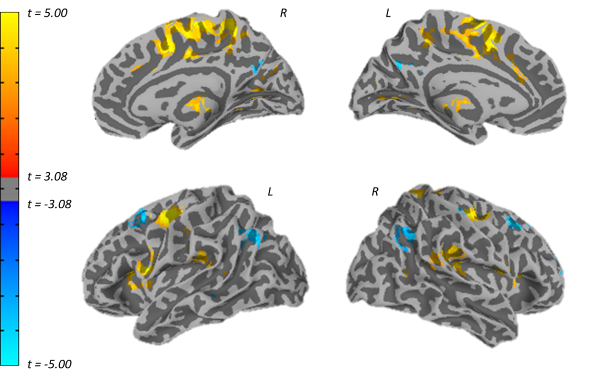


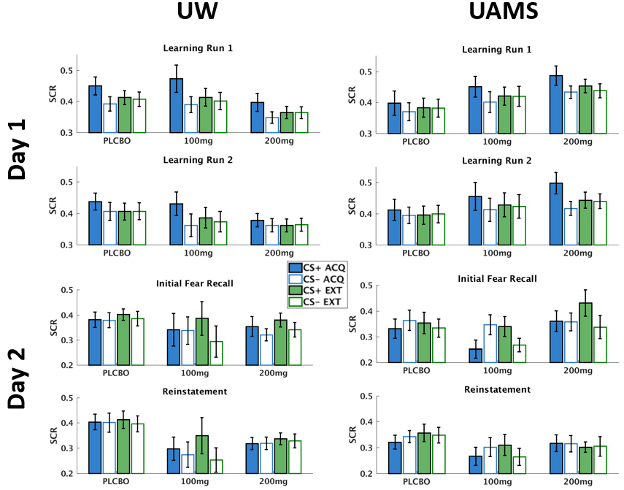


**Supplemental Figure 7.** Comparison of skin conductance responses for Day 1 learning (top row) and Day 2 recall across the UW (left column) and UAMS (right column) sites.

**Supplemental Figure 8.** Comparison of insula / inferior frontal gyrus (IFG) network activation during Day 2 recall between the UW (left) and UAMS (right) sites.


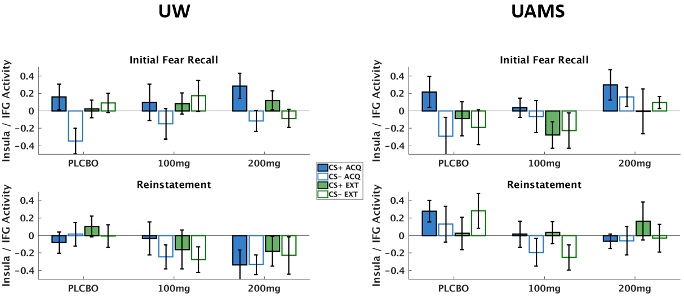


Supplemental References

1 Haaker J, Gaburro S, Sah A, Gartmann N, Lonsdorf TB, Meier K *et al.* Single dose of l-dopa makes extinction memories context-independent and prevents the return of fear. *Proc Natl Acad Sci* 2013; **110**: E2428–E2436.

2 Raij T, Nummenmaa A, Marin M-F, Porter D, Furtak S, Setsompop K *et al.* Prefrontal Cortex Stimulation Enhances Fear Extinction Memory in Humans. *Biol Psychiatry* 2018; **84**: 129–137.

3 Garfinkel SN, Abelson JL, King AP, Sripada RK, Wang X, Gaines LM *et al.* Impaired Contextual Modulation of Memories in PTSD: An fMRI and Psychophysiological Study of Extinction Retention and Fear Renewal. *J Neurosci* 2014; **34**: 13435–13443.

4 Bach DR. A head-to-head comparison of SCRalyze and Ledalab, two model-based methods for skin conductance analysis. *Biol Psychol* 2014; **103**: 63–68.

5 Bach DR, Flandin G, Friston KJ, Dolan RJ. Time-series analysis for rapid event-related skin conductance responses. *J Neurosci Methods* 2009; **184**: 224–234.

6 Bach DR, Flandin G, Friston KJ, Dolan RJ. Modelling event-related skin conductance responses. *Int J Psychophysiol Off J Int Organ Psychophysiol* 2010; **75**: 349–356.

7 Bach DR, Friston KJ, Dolan RJ. An improved algorithm for model-based analysis of evoked skin conductance responses. *Biol Psychol* 2013; **94**: 490–497.

8 Bach DR, Friston KJ. Model-based analysis of skin conductance responses: Towards causal models in psychophysiology. *Psychophysiology* 2013; **50**: 15–22.

9 Gerster S, Namer B, Elam M, Bach DR. Testing a linear time invariant model for skin conductance responses by intraneural recording and stimulation. *Psychophysiology* 2017. doi:10.1111/psyp.12986.

10 Staib M, Castegnetti G, Bach DR. Optimising a model-based approach to inferring fear learning from skin conductance responses. *J Neurosci Methods* 2015; **255**: 131–138.

11 Bach DR, Tzovara A, Vunder J. Blocking human fear memory with the matrix metalloproteinase inhibitor doxycycline. *Mol Psychiatry* 2017. doi:10.1038/mp.2017.65.

12 Eklund A, Nichols TE, Knutsson H. Cluster failure: Why fMRI inferences for spatial extent have inflated false-positive rates. *Proc Natl Acad Sci* 2016; **113**: 7900–7905.

13 Molapour T, Golkar A, Navarrete CD, Haaker J, Olsson A. Neural correlates of biased social fear learning and interaction in an intergroup context. *NeuroImage* 2015; **121**: 171–183.

14 Morriss J, Hoare S, van Reekum CM. It’s time: A commentary on fear extinction in the human brain using fMRI. *Neurosci Biobehav Rev* 2018; **94**: 321–322.

15 Gerlicher AMV, Tüscher O, Kalisch R. Dopamine-dependent prefrontal reactivations explain long-term benefit of fear extinction. *Nat Commun* 2018; **9**: 4294.
